# Cytomegalovirus (CMV) genotype in allogeneic hematopoietic stem cell transplantation

**DOI:** 10.1186/1471-2334-13-310

**Published:** 2013-07-10

**Authors:** Débora C Dieamant, Sandra HA Bonon, Renata MB Peres, Claudia RC Costa, Dúlcinéia M Albuquerque, Eliana CM Miranda, Francisco JP Aranha, Gislaine Oliveira-Duarte, Virginio CA Fernandes, Carmino A De Souza, Sandra CB Costa, Afonso C Vigorito

**Affiliations:** 1 Department of Clinical Medicine, Faculty of Medical Sciences, University of Campinas, Rua Vital Brazil, 251, ZIP Code: 13083-888 Campinas, SP, Brazil; 2 Hematopoietic Stem Cell Transplantation Unit of the University of Campinas Teaching Hospital, University of Campinas, Campinas, SP, Brazil

**Keywords:** CMV genotyping, Nested-PCR, Antigenemia, GVHD, qPCR, RFLP

## Abstract

**Background:**

Based on sequence variation in the *UL55* gene that encodes glycoprotein B (*gB*), human cytomegalovirus (CMV) can be classified into four *gB* genotypes. Previous studies have suggested an association between CMV gB genotype and clinical outcome in patients who underwent an allogeneic hematopoietic stem cell transplant (HSCT). The goals of this study were identify patients with active infection caused by CMV in recipients of HSCT; determine the prevalence of CMV genotypes in the study group; correlate genotype with CMV disease, acute GVHD and overall survival.

**Methods:**

The diagnosis of active CMV infection after allogeneic HSCT was detected by antigenemia (AGM) and/or nested-PCR (N-PCR). Positive samples from patients with active CMV infection were submitted to genotyping using N-PCR to amplify a region of *UL55*, followed by restriction analysis based on *Hin*fI and *Rsa*I digestion. Real-time PCR (qPCR) was used to determine the viral load during active CMV infection and antiviral treatment.

**Results:**

Sixty-three allogeneic HSCT recipients were prospectively evaluated; 49/63 (78%) patients were infected with CMV genotypes – gB1 19/49 (39%), gB2 17/49 (35%), gB3 3/49 (6%), gB4 7/49 (14%) – and 3 (6%) had mixed CMV genotypes (gB1 + gB3, gB1 + gB4 and gB2 + gB4). Characterized by gastrointestinal disease, CMV disease occurred in 3/49 (6.1%) patients, who had CMV gB3 genotype. These gB3 genotype patients presented an increasing AGM number, mean 125 (± 250) (*P* = 0.70), and qPCR copies/ml, mean 37938 (SD ± 50542) (*P* = 0.03), during antiviral treatment, when compared with other CMV genotypes. According to CMV genotypes, stratified overall survival was 55% for gB1, 43% for gB2; 0% for gB3 and 57% for gB4 (*P* = 0.03).

**Conclusions:**

One of the restrictions of the presented study was the low number of CMV gB sub-cohorts). However, we demonstrated that the frequency of active CMV infection in this HSCT population was high, and the most prevalent genotype in these patients with active CMV infection was gB1 and gB2 genotype (74%). In Brazil, HSCT recipients seem to carry mainly gB1 and gB2 CMV genotype.

## Background

Cytomegalovirus (CMV) remains the most important cause of serious viral infections in allogeneic hematopoietic stem cell transplant (HSCT) recipients [1]. CMV glycoprotein B (gB) is the major CMV envelope glycoprotein and it is encoded by UL55 gene. CMV gB has been implicated in host cell entry, cell-to-cell viral transmission and fusion of infected cells [2-4]. Chou and Dennison (1991) devised a method of CMV genotyping based on UL55 gene nucleotide sequence that encodes a variable region encompassing the protease cleavage site. They found that there were *HinfI* and *RsaI* restriction sites between nucleotides 1344 and 1440. Amplification of this region, using polymerase chain reaction (PCR) followed by restriction analysis, demonstrated the existence of four different gB genotypes [5]. Since gB has been implicated in host cell penetration, it is possible that four types differ with respect to tissue tropism and virulence. Many studies have attempted to find a correlation between gB genotype and the occurrence of CMV-associated disease in immunocompromised patients; however, it remains unclear whether certain gB genotypes are associated with an increased frequency of disease [6]. There are few references about functional differences that may exist among various CMV strains. It was reported that the existence of CMV variants played an important role in the pathogenesis of diseases, as these variants affected several genes that might be responsible for different diseases related to active CMV infection [7-10]. Recently, comparative sequence studies have been used to define the extent of interstrain variation in selected coding regions of the CMV genome. Clinical CMV isolates were found to adopt one of four gB sequence configurations at certain variable *loci*, and a genotyping scheme was proposed [11-13]. Although gB genotypes display significant amino acid variations in their variable domains, including changes that affect glycosylation sites, the functional consequences of these variations have not yet been explored [14-24].

This study aimed to prospectively analyze gB gene of CMV in allogeneic hematopoietic stem cell transplant (HSCT) recipients with human active CMV infection to determine the distribution of gB genotypes and their possible impact on overall survival, acute GVHD and CMV disease.

## Methods

### Patients and samples

This study comprised 63 adult patients with malignant and nonmalignant hematological diseases, who underwent a myeloablative or nonmyeloablative allogeneic HSCT at the Hematopoietic Stem Cell Transplantation Unit of the University of Campinas Teaching Hospital, with related or not related HLA identical donors and graft source from bone marrow or peripheral blood. The blood products used were neither screened for CMV antibody nor filtered to depleted leukocytes, although all had been irradiated. The conditioning regimens and GVHD prophylaxis were selected according to ongoing protocols at the University Hospital. These patients were prospectively monitored for active CMV infection from March 2007 to May 2010, using antigenemia (AGM) assay, nested-polymerase chain reaction (N-PCR) in leukocytes, and real-time polymerase chain reaction (qPCR). Patients with active CMV infection results were treated with preemptive ganciclovir. CMV genotypes of all positive patients were obtained by N-PCR and confirmed by sequencing, using primers from the glycoprotein B (gB) region of CMV (UL55) and restriction enzyme analysis (RFLP) with *HinfI* and RsaI enzymes. Real-time polymerase chain reaction (qPCR) assay was used to quantify the CMV load during active CMV infection and antiviral preemptive treatment. The protocol was designed in accordance with the requirements for research involving human subjects in Brazil and approved by the Institutional Review Board. Informed consent was obtained from all patients. Clinical characteristics of the study subjects are summarized in Table 1.

**Table 1:** Patient and transplant characteristics

**Characteristics**	** n = 63**
**Patient age, median (range), y**	42 (16–65)
**Diagnosis at transplant, no. (%)**	
***Malignant diseases***	
Acute myeloid leukemia	23 (36.5%)
Chronic myeloid leukemia	9 (14.3%)
Acute lymphoblastic leukemia	9 (14.3%)
Chronic lymphocytic leukemia	4 (6.3%)
Multiple myeloma	2 (3.2%)
Non-Hodgkin lymphoma	3 (4.8%)
Hodgkin lymphoma	2 (3.2%)
Myelodysplastic syndrome	4 (6.3%)
***Non-malignant diseases***	
Severe aplastic anemia	4 (6.3%)
Paroxysmal nocturnal hemoglobinuria	3 (4.8%)
**Donor age, median (range), y**	39 (6–65)
**Patient gender (donor/recipient), n. (%)**	
Male/male	23 (36.5%)
Male/female	10 (15.9%)
Female/male	13 (20.6%)
Female/female	17 (27%)
**Donor type, no. (%)**	
HLA-identical related	61 (96.8%)
HLA-matched unrelated	2 (3.2%)
**Conditioning regimen, no. (%)**	
High dose	46 (73%)
Low dose	17 (27%)
**Source of stem cells, no. (%)**	
Bone marrow	23 (36.5%)
Mobilized blood	40 (63.5%)
**GVHD prophylaxis, no. (%)**	
Cyclosporine plus methotrexate	49 (77.7%)
Cyclosporine plus mycophenolate mofetil	14 (22.3%)
**Acute GVHD, no. (%)**	17 (27%)
Grade 0-I	46 (73%)
Grade II-IV	17 (27%)
**Donor/recipient CMV serologic status, n° (%)**	
IgG +/IgG+	55 (87.2%)
IgG +/IgG -	3 (4.8%)
IgG -/IgG -	2 (3.2%)
IgG-/igG+	2 (3.2%)
Not determined	1 (1.6%)

*GVDH* graft-versus-host disease, *CMV* cytomegalovirus.

The study was approved by the National Research Ethics (CONEP) of Brazil (680/2006).

### Definitions

Active CMV infection was defined based on at least one of the following criteria: [1] one or more positive cells in the AGM assay, and [2] two or more consecutive positive N-PCR results. For the diagnosis of CMV disease, the active infection had to be accompanied by clinical symptoms and histopathological identification of CMV [25]. Recurrence of CMV infection was defined as active CMV infection occurring after negative N-PCR and/or AGM assays, following treatment of the initial episode of infection. Late active CMV infections and diseases were defined as those occurring more than 100 days after transplant.

### Antigenemia assay

AGM assay was done at least once a week after engraftment, according to Bonon et al., 2005, with some modifications. EDTA-treated blood samples were fractionated by erythrocyte lyses. Granulocytes were then centrifuged to prepare cytospin slides (2 × 10^5^ granulocytes per slide). After air-drying and fixing the slides in formaldehyde, they were immunostained using the well-defined C10/C11 antibody cocktail to detect the CMV lower matrix phosphoprotein (*pp65*), an early antigen in virus replication, which is abundantly present in antigen-positive polymorphonuclear cells. The CMV Brite™ Turbo Kit (Iq Products) is a rapid new version of the first FDA registered immunofluorescence antigenemia kit for in vitro CMV diagnosis. Slides were made in duplicate [26].

### Nested polymerase chain reaction (N-PCR)

CMV DNA in blood specimens was detected by nested PCR using the primers described by Demmler et al. and Shibata et al. (1988). Briefly, leukocytes remaining from the CMV antigenemia assay were lysed and the DNA was precipitated. The primers were selected from the MIE region of CMV-AD169. The size of the PCR amplification products was 159 base pairs. The same protocol was used to amplify the human b-globin gene sequence to guarantee the quality of the extracted DNA [27,28].

### Cytomegalovirus viral load assay

#### Real-time PCR

PCR primer and probe sequences were selected from the US17 region of CMV AD169. The real-time protocol was according to Peres et al., 2010. The forward and reverse CMV primers were 5′ GAAGGTGCAGGTGCCCTG 3′ and 5′ GTGTCGACGAACGACGTACG 3′, respectively. The Taq Man probe selected between both primers was fluorescence labeled with 6-carboxyfluorescein at the 5′ end as the reporter dye and 6- carboxytetramethylrhodamine at the 3′ end as the quencher (5′FAM ACGGTGCTGTAGACCCGCATACAAA TAMRA3′).

A search of databases indicated that neither the primers nor the probes shared significant homology with any known nucleotide sequence except of CMV. The real-time PCR was performed with a mixture containing: 3 mM MgCl_2_; 10 μM dATP, dCTP, dGTP, dTTP; 5 U/μl of Platinum *Taq* (Invitrogen), 60 ng DNA templates, 150 nM of forward and reverse primers (CMVUS17F-CMVUS17R for CMV detection) and 2 μM of the specific Taq Man CMV probe (PE Applied Biosystems). The single PCR was performed in 96-well microliter plates under the following conditions: 1 cycle at 50°C for 2 minutes, 95°C for 10 minutes and 45 cycles at 95°C for 15 seconds and 60°C for 1 minute. The ß-actin gene amplification was performed under the same PCR conditions described above for the reaction control, using 2 μM ß-actin probe (FAM™ Probe), 3 μM ß-actin forward primer, and 3 μM ß-actin reverse primer (TaqMan® ß-actin detection reagents - Applied Biosystems) [29].

### Amplification of gB gene by nested-PCR

Oligonucleotide primers used for PCR amplification were chosen in a region of high sequence variability in the CMV gB gene, as previously published by Chou and Dennison (1991), and were synthesized commercially (Invitrogen, by Life Technologies, Brazil). The first and the second rounds of amplification were carried out in a total volume of 50 μl, using 200 ng DNA extract (1st) and 1 μl PCR product (2nd) and 49 μl PCR mix (10 mM Tris pH 8.3, 50 mM KCl, 2 mM MgCl2, 200 mM of each dNTPs, 1.25 U of recombinant *Taq DNA polymerase* and 0.4 mM of each primer (Invitrogen, by Life Technologies, Brazil). After amplification, 5 μl of the amplified product were electrophoresed on 2% agarose gel (Gibco-BRL, Grand Island, NY) containing ethidium bromide, and the gel was photographed under UV illumination. The AD169 strain was used as a positive control; an uninfected DNA sample or water was used as a negative control [5].

### CMV gB genotyping with PCR-RFLP analysis and sequence analyses

Approximately 10 μl of nested PCR product were digested at 37°C overnight, using 1 U of the restriction enzymes, *Rsa I* and *Hinf I* (Gibco-BRL). Sequences were analyzed on a 2% agarose 1000 gel (Gibco-BRL). The four types of gB were distinguished by their different patterns of fragment lengths, as described [5].

To determine the sequences of the CMV gB genotype samples, the PCR products were purified using the PCR purification kit (Quiagen) and subcloned into the pGEM-T vector (Promega, Madison, WI, USA). Sequences were obtained using the ABI 310 genetic analyser (Applied Biosystems, Bedford, MA, USA) with proper primers, and they were aligned with known CMV variants in GenBank afterward. The sequences of gB1, gB2, gB3 and gB4 were very similar to GenBank M60927, M60931, M60930 and M60926 respectively.

### Statistical analysis

The descriptive analysis summarized patient sociodemographic characteristics, as well as transplant, GVHD and CMV serologic status of donors and receptors. The Fisher’s and Kruskal-Wallis tests were applied for categorical and continuous variables, respectively. The Kaplan-Meier method was used to estimate overall survival. Stratified overall survival, according to CMV genotypes, was compared with the log-rank test. Death by any cause was considered as event. The gB1 + gB3 mixture was clustered into genotype gB3 for statistical purpose, since these strains had the same behavior in the study.

The other two mixtures detected, gB1 + gB4 and gB2 + gB4, were not grouped, as they did not identify any pattern of behavior. The *p*-value < 0.05 was considered significant. The statistical analyses were performed using the software SPSS 14 (Statistical Package for Social Sciences).

## Results

Among the 63 patients, 49 (78%) had active CMV infection detected by AGM and/or N-PCR tests. Active CMV infection occurred within a median time of 38 days (1–150) after the transplant. The median time to detect active CMV infection by AGM and N-PCR was 46 (14–150) and 34 days (1–140), respectively. The incidence of active CMV infections is summarized in Table 2.

**Table 2:** Incidence of active CMV infection

**Patients**	**n = 63**
Positive *N-PCR* and/or AGM, n (%)	49 (78%)
Median time, days (range)	38 (1–150)
Positive *N-PCR*, n (%)	49 (78%)
Median time, days (range)	34 (1–140)
Positive *AGM*, n (%)	37 (58.7%)
Median time, days (range)	46 (14–150)

*N-PCR* nested polymerase chain reaction, AGM antigenemia.

### CMV gB genotype

Forty-nine patients with active CMV infection were submitted to CMV genotyping using RFLP. Nineteen out of 49 (39%) recipients had gB1 genotype; 17 out of 49 (35%), gB2; 3 out of 49 (6%), gB3; and 7 out of 49 (14%), gB4. There were three patients (6%) who had a combination of two different CMV genotypes (gB1 + gB3, gB1+ gB4 and gB2 + gB4).

Eight samples (two with gB1, two with gB2, two with gB3 and two with gB4) and three mixed CMV genotype samples were sequenced. The patients’ CMV sequences were 98-99% identical to CMV sequences in GenBank when compared.

### CMV disease, acute GVHD and gB genotype

Among the 49 patients who had active CMV infection, three (6%) developed CMV disease manifested as gastrointestinal disease, two had gB3 and one had a mixture of gB1 + gB3 strains.

The active CMV infection recurrence detected by HCMV antigenemia and/or nested PCR occurred in 9 out of 49 (18%) patients within a median of 92 days (61–138), with the same infecting strain caused by the first active CMV infection. The recurrence occurred in 1 out of 9 (11%) cases with gB1, in 1 out of 9 (11%) with gB2, in 3 out of 9 (33%) with gB3, and in 4 out of 9 (44%) with gB4.

Seventeen out of 49 (35%) patients with CMV active infection developed grade II-IV acute GVHD. Among the patients with acute GVHD, 12 out of 17 (70.6%) had acute GVHD before diagnosis of active CMV infection; however, 5 out of 17 (29.4%) patients had active CMV infection before acute GVHD. Diagnosis of active CMV infection occurred within a median of 20 days [6-35] after diagnosis of acute GVHD, and acute GVHD occurred within a median of 45 days (27–59) after active CMV infection. The frequency of II-IV acute GVHD, according to CMV gB genotype, showed that 8 out of 19 gB1 patients (42%), 1 out of 17 gB2 patients (6%), 3 out of 7 gB4 patients (43%) had aGVHD, and all 4 gB3 patients, including the mixture gB1 + gB3, had aGVHD (*P* = 0.008).

### CMV DNA load at onset of active CMV infection and during preemptive treatment monitoring

At the active CMV infection onset, gB3 genotype presented the highest number of AGM-positive, mean 251 (SD ± 499), and gB2 the highest qPCR copies/ml, mean 1733 (SD ± 6272). However, the difference between the four genotypes for either AGM or qPCR was not significant (AGM – *p-value* = 0.73; qPCR - *p-value* = 0.13). These results are summarized in Table 3.

**Table 3:** Results of AGM and viral load by qPCR at active CMV infection and during preemptive treatment monitoring

**Genotypes**		**Median**	**Range**	**Mean**	**SD**	Mean*	**SD**
**gB1**	**AGM** (n cells)	3	0-38	8	± 12	-	-
	**qPCR** (copies/ml)	92	15-804	388	± 864	2.05	± 0.68
**gB2**	**AGM** (n cells)	3	0-10	3	± 3	-	-
	**qPCR** (copies/ml)	23	3-26012	1733	± 6272	1.72	± 1.07
**gB3**	**AGM** (n cells)	2	1-1000	251	± 499	-	-
	**qPCR** (copies/ml)	12	3-753	195	± 372	1.37	± 1.05
**gB4**	**AGM** (n cells)	1	0-70	11	± 26	-	-
	**qPCR** (copies/ml)	351	13-2283	675	± 892	2.32	± 0.84

*AGM* antigenemia, *qPCR* real-time polymerase chain reaction.

*Mean qPCR in logarithm base 10; n = number of *pp65* CMV positive cells/2 × 10^5^; SD = standard deviation; AGM – *p-value* = 0.73; qPCR - *p-value* = 0.13 (kruskal-WallisTest).

**Figure 1:**
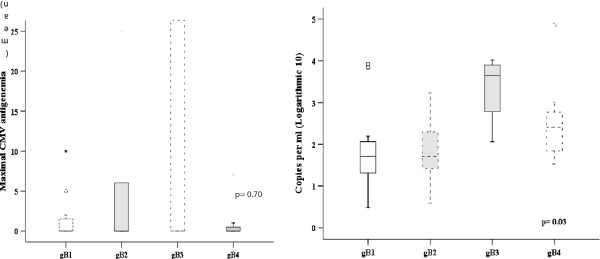
Antigenemia and viral load by qPCR in the monitoring of preemptive antiviral treatment stratified by genotypes.

In spite of preemptive treatment for active CMV infection, three out of four gB3 patients developed gastrointestinal disease. In two out of those three patients, CMV replication occurred after onset of grade II-IV acute GVHD. Interesting, during preemptive treatment, these gB3 genotype patients presented an increasing AGM number, mean 125 (± 250) (*p* = 0.70), and qPCR copies/ml, mean 37938 (SD ± 50542), when compared with other CMV genotypes (*p* = 0.03). These results are presented in Table 4 and Figure 1.

**Table 4:** Results of AGM and viral load by qPCR in the monitoring of preemptive antiviral treatment stratified by genotypes

**Genotypes**		**Median**	**Range**	**Mean**	**SD**	Mean*	**SD**
**gB1**	**AGM** (n cells)	0	0-200	12	± 46	-	-
	**qPCR** (copies/ml)	261	15-8358	1247	± 2352	2.51	± 0.76
**gB2**	**AGM** (n cells)	0	0-500	63	± 165	-	-
	**qPCR** (copies/ml)	359	5-26012	4088	± 8537	2.45	± 1.16
**gB3**	**AGM** (n cells)	0	0-500	125	± 250	-	-
	**qPCR** (copies/ml)	20751	115-110136	37938	± 50542	3.86	± 1.31
**gB4**	**AGM** (n cells)	0	0-7	1	± 3	-	-
	**qPCR** (copies/ml)	4690	46-73084	15758	± 26024	3.41	± 1.23

*AGM* antigenemia, *qPCR* real-time polymerase chain reaction.

*Mean qPCR transformed in logarithmic base 10; n = number of *pp65* CMV positive cells/2 × 10^5^; SD = standard deviation. Antigenemia – *P-value* = 0.70; qPCR - *P-value* = 0.03 (kruskal-WallisTest).

### Overall survival and CMV gB genotype

Among the 49 patients with active CMV infection, 25 (51%) died. The distribution of causes of death were as follows: relapse, 10 out of 25 (40%); bacterial or fungal infection, 10 out of 25 (40%); GVHD, 2 out of 25 (8%); cardiac toxicity, 1 out of 25 (4%); pulmonary hemorrhage, 1 out of 25 (4%); CMV disease, 1 out of 25 (4%).

**Figure 2:**
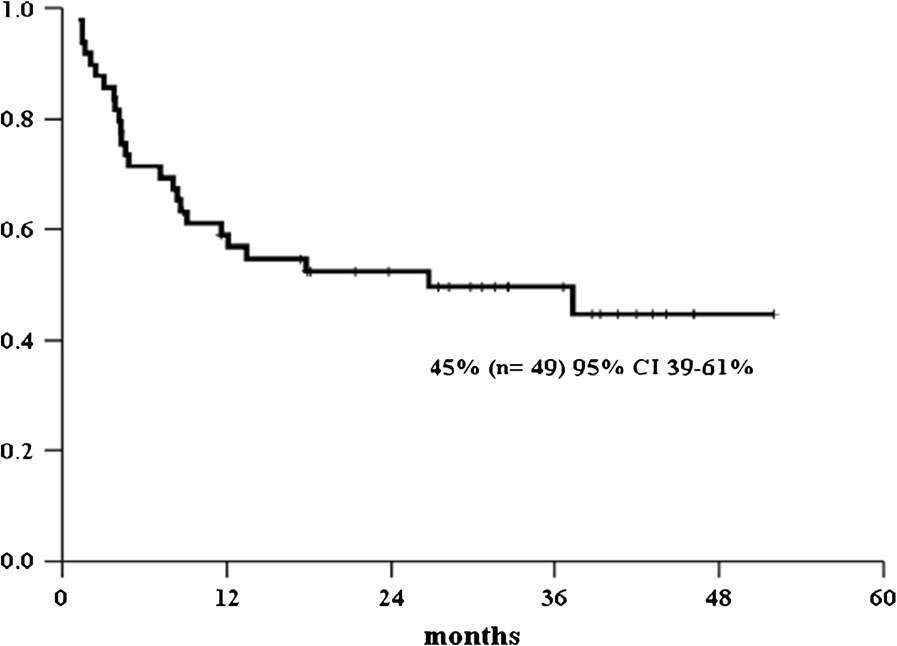
Overall survival.

**Figure 3:**
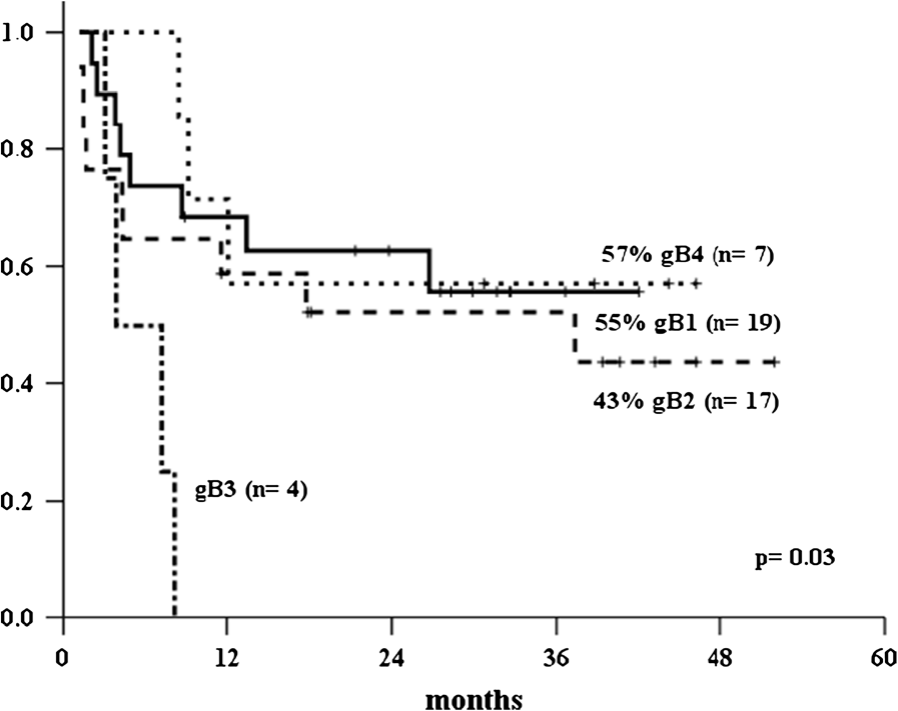
Overall survival by genotype.

After a median follow-up of 17 months [1-52], the overall survival for patients with active CMV infection was 45% (95% CI 39-61%); whereas the stratified overall survival, according to CMV genotypes, was 55% for gB1, 43% for gB2; 0% for gB3 and 57% for gB4 (*p* = 0.03), as shown in Figures 2 and 3.

## Discussion

In this cohort of Brazilians patients with active CMV infection, the most frequent genotypes were gB1 and gB2 (74%). Moreover, all patients who had CMV gastrointestinal disease, higher viral load during preemptive antiviral treatment, acute GVHD grade II-IV and worse survival were gB3 genotype.

The high incidence of active CMV infection detected either by N-PCR or AGM in our population was comparable to prior reports and they were equally effective for diagnosis of active infection and disease [26,30-32]. Although the proportion of CMV seronegative donors or recipients has been small, almost all positive N-PCR and AGM results were from CMV seropositive patients.

According to previously described CMV envelope glycoprotein genotypes, we were able to confirm that CMV gB genotyping could reliably identify one of four established gB genotypes, using two restriction endonucleases – HinfI and RSAI – to digest the PCR-amplified variable region of UL55, encoding the protease cleavage site [5,33]. GB1 and gB2 were the most frequent genotypes in this study, and they occurred in similar proportions (39% and 35%, respectively). These results are in line with other studies, including one from our group performed in a Brazilian pediatric renal and hematopoietic stem cell transplantation cohort [7,34-37], but not with a report from Chinese HSCT patients, where gB1 and gB3 were the prevalent genotypes [38,39]. Furthermore, in our population, the proportion of mixed genotypes was rare (6%), that is comparable to recent reports which showed ranges from 5% to 25% [6,38,40].

CMV gB genotype may be an important determinant of viral virulence because gB has been implicated in several essential steps in CMV pathogenesis, such as virus entry, cell fusion, and cell-to-cell spread. The virulence of different CMV strains may be an important factor in the occurrence of CMV disease because of genetic variation in genes that are involved in host cell penetration, tissue tropism, or replication, and polymorphism in the viral genome may play an important role [40-42].

Outcomes of different CMV genotypes with clinical manifestations are conflicting. CMV gB3 and gB4 were reported to be associated with myelosuppression in HSCT patients [35], gB3 with high incidence of pneumonitis [38], and gB1 with invasive disease in solid organ transplantation [36,38]. A recent Brazilian study involving AIDS patients demonstrated that gB2 genotype was associated with worse prognosis [43]. In prior studies, mixed CMV genotypes were observed to be associated with a high prevalence of CMV disease in solid-organ transplant patients [6,44,45], but in our study we did not find these results.

Neither cases of myelossupression and pneumonitis, nor a high prevalence of CMV disease in mixed CMV genotypes were seen in our data, but intriguing all cases of CMV disease were associated with the gastrointestinal tract, and all of them had genotype gB3. Hence, gB3 genotype might confer a specific virulence advantage for that genotype in our cohort. Although some associations have been described so far between a certain virus subtype and the development of individual disease these analyses were greatly complicated by the huge genomic background of CMV, by the large variety of individual host-virus relations and by differences in the geographic or demographic subtype distribution [46].

Acute GVHD and CMV replication are pathogenetically associated. In this report, most patients had GVHD before the onset of CMV infection, confirming what several studies have already shown that acute GVHD and its treatment put patients at risk for CMV replication [43,47-49]. In contrast, the role of CMV replication as a cause of acute GVHD is controversial. One recent small study found no effect of CMV replication on subsequent development of acute GVHD [50]. Others demonstrated the reciprocal finding that patients are at significant risk of developing acute GVHD during CMV replication [43,51].

Torok-Storb et al. [35] reported that CMV gB3 was associated with a reduced risk of GVHD in HSCT patients. Wu et al. [38] failed to demonstrate an association of B genotypes with GVHD. We found a different distribution of II-IV acute GVHD according to gB genotypes, and all gB3 genotypes were involved with acute GVHD, highlighting a possible association of that genotype with acute GVHD.

We observed that the mean load viral was higher in gB2 at the diagnosis; however, during the preemptive antiviral treatment, the viral load detected, either by qPCR and AGM, showed an increase in gB3 genotype. Clearly, genotype gB3 showed a different behavior when compared with other genotypes, leading to believe that this might be associated with a more severe and uncontrolled infection that caused all cases of gB3 gastrointestinal CMV disease and a worse survival in our population.

Perhaps, not gB3 genotype per se but its low frequency in the patient’s cohort may be associated with bad outcome of the CMV infection. If this genotype is rare in this region, higher is the probability of primo infection in this population during the transplantation, that is associated with insufficient or delayed immune response, prolonged high level of virus replication and higher risk of CMV disease and related complications.

## Conclusion

One of the restrictions of the presented study was the low number of CMV gB sub- cohorts). However, we demonstrated that the frequency of active CMV infection in this HSCT population was high and the most prevalent genotype in these patients with active CMV infection was gB1 and gB2 genotype (74%). In Brazil, HSCT recipients seem to carry mainly gB1 and gB2 CMV genotype.

## Competing interests

The authors declare that they have no competing interests.

## Authors’ contribution

ACV study design, collected data, analyzed data and wrote the paper; DCD study design, collected data, analyzed the data and wrote the paper; ECMM performed the statistical analysis; SHB, RMBP, CRCC, DMA updated the collected the data for the study; FJPA, GO-D, VCAF, CADS reviewed the manuscript; SCBC study design and reviewed the manuscript. All authors read and approved the final manuscript.

## Pre-publication history

The pre-publication history for this paper can be accessed here:

http://www.biomedcentral.com/1471-2334/13/310/prepub
